# Supplementary material for: Multiple-instance learning of somatic mutations for the classification of tumour type and the prediction of microsatellite status
Source: Nat Biomed Eng. 2023 Nov 2;8(1):57–67. doi: 10.1038/s41551-023-01120-3 (PMC10805698; doi:10.1038/s41551-023-01120-3)
Supplement: Supplementary file 2 — Reporting Summary [file 41551_2023_1120_MOESM2_ESM.pdf]

nature portfolio

Corresponding author(s): Alexander S. Baras

Last updated by author(s): Sep 20, 2023

Reporting Summary

Nature Portfolio wishes to improve the reproducibility of the work that we publish. This form provides structure for consistency and transparency in reporting. For further information on Nature Portfolio policies, see our Editorial Policies and the Editorial Policy Checklist.

Statistics

For all statistical analyses, confirm that the following items are present in the figure legend, table legend, main text, or Methods section.

n/a	Confirmed	
☒	☐	The exact sample size (*n*) for each experimental group/condition, given as a discrete number and unit of measurement
☒	☐	A statement on whether measurements were taken from distinct samples or whether the same sample was measured repeatedly
☒	☐	The statistical test(s) used AND whether they are one- or two-sided<br>*Only common tests should be described solely by name; describe more complex techniques in the Methods section.*
☒	☐	A description of all covariates tested
☒	☐	A description of any assumptions or corrections, such as tests of normality and adjustment for multiple comparisons
☒	☐	A full description of the statistical parameters including central tendency (e.g. means) or other basic estimates (e.g. regression coefficient) AND variation (e.g. standard deviation) or associated estimates of uncertainty (e.g. confidence intervals)
☒	☐	For null hypothesis testing, the test statistic (e.g. *F*, *t*, *r*) with confidence intervals, effect sizes, degrees of freedom and *P* value noted<br>*Give P values as exact values whenever suitable.*
☒	☐	For Bayesian analysis, information on the choice of priors and Markov chain Monte Carlo settings
☒	☐	For hierarchical and complex designs, identification of the appropriate level for tests and full reporting of outcomes
☒	☐	Estimates of effect sizes (e.g. Cohen's *d*, Pearson's *r*), indicating how they were calculated

Our web collection on statistics for biologists contains articles on many of the points above.

Software and code

Policy information about availability of computer code

Data collection	No new data collection was performed.
Data analysis	Python modules used: logomaker==0.8, matplotlib==3.5.1, numpy==1.22.2, pandas==1.4.1, scikit-learn==1.0.2, tensorflow==2.7.0, biopython==1.78, pyranges==0.0.115, scikit-optimize==0.9.0.<br><br>The code that we developed and used in this study is available at https://doi.org/10.5281/zenodo.8083498.

For manuscripts utilizing custom algorithms or software that are central to the research but not yet described in published literature, software must be made available to editors and reviewers. We strongly encourage code deposition in a community repository (e.g. GitHub). See the Nature Portfolio guidelines for submitting code & software for further information.

Data

Policy information about availability of data

All manuscripts must include a data availability statement. This statement should provide the following information, where applicable:

- Accession codes, unique identifiers, or web links for publicly available datasets
- A description of any restrictions on data availability
- For clinical datasets or third party data, please ensure that the statement adheres to our policy

All data used in this publication are publicly available. The MC3 MAFs are from ref. 32, and the MSI PCR labels are available from the cBioPortal, TCGAbiolinks or

individual publications33–36. The UCSC simpleRepeat.txt was downloaded from http://hgdownload.cse.ucsc.edu/goldenPath/hg19/database/simpleRepeat.txt.gz. The Broad coverage WIGs are available at https://www.synapse.org/#!Synapse:syn21785741. MANTIS values are from ref. 37. The MAF for the ICGC PCAWG samples was obtained from https://dcc.icgc.org/releases/PCAWG/consensus_snv_indel, and the MAF for the TCGA PCAWG samples was obtained from https://icgc.bionimbus.org/files/0e8a845d-a4f4-40bc-890b-5472702d087c.

Research involving human participants, their data, or biological material

Policy information about studies with human participants or human data. See also policy information about sex, gender (identity/presentation), and sexual orientation and race, ethnicity and racism.

Reporting on sex and gender	No sex or gender analyses were performed.
Reporting on race, ethnicity, or other socially relevant groupings	No such analyses were performed.
Population characteristics	Data from the PCAWG and MC3 working groups were used in this study, with patients from the ICGC and TCGA cohorts. The patient recruitment is described in 'Pan-cancer analysis of whole genomes' (Nature, 2020), and in 'Cell-of-Origin Patterns Dominate the Molecular Classification of 10,000 Tumors from 33 Types of Cancer' (Cell, 2018).
Recruitment	Not applicable.
Ethics oversight	Not applicable.

Note that full information on the approval of the study protocol must also be provided in the manuscript.

Field-specific reporting

Please select the one below that is the best fit for your research. If you are not sure, read the appropriate sections before making your selection.

☒ Life sciences ☐ Behavioural & social sciences ☐ Ecological, evolutionary & environmental sciences

For a reference copy of the document with all sections, see nature.com/documents/nr-reporting-summary-flat.pdf

Life sciences study design

All studies must disclose on these points even when the disclosure is negative.

Sample size	For tumour classification, we set a minimal sample size, the selection of which was arbitrary, but was influenced by the need to have enough samples for stratified K-fold training.
Data exclusions	Variants which did not fall within the corresponding coverage WIGs were excluded.
Replication	In the case of MSI, that analysis was performed across three different iterations of our model over the last 3 years, along with slightly different approaches to data processing, and the results were highly similar each time. In the case of cancer classification we performed the classification with both project codes and NCIt labels, achieving highly similar results.
Randomization	Sklearn was used for randomization, with stratification.
Blinding	Not applicable.

Reporting for specific materials, systems and methods

We require information from authors about some types of materials, experimental systems and methods used in many studies. Here, indicate whether each material, system or method listed is relevant to your study. If you are not sure if a list item applies to your research, read the appropriate section before selecting a response.

Materials & experimental systems

n/a	Involved in the study
☒	☐ Antibodies
☒	☐ Eukaryotic cell lines
☒	☐ Palaeontology and archaeology
☒	☐ Animals and other organisms
☒	☐ Clinical data
☒	☐ Dual use research of concern
☒	☐ Plants

Methods

n/a	Involved in the study
☒	☐ ChIP-seq
☒	☐ Flow cytometry
☒	☐ MRI-based neuroimaging
